# Baseline plasma corticosterone, haematological and biochemical results in nesting and rehabilitating loggerhead sea turtles (*Caretta caretta*)

**DOI:** 10.1093/conphys/cov003

**Published:** 2015-03-02

**Authors:** Jennifer E. Flower, Terry M. Norton, Kimberly M. Andrews, Steven E. Nelson, Clare E. Parker, L. Michael Romero, Mark A. Mitchell

**Affiliations:** 1 Department of Veterinary Clinical Medicine, College of Veterinary Medicine, University of Illinois, Urbana, IL 61802, USA; 2 Georgia Sea Turtle Center, Jekyll Island Authority, Jekyll Island, GA 31527, USA; 3 Department of Biology, Tuft's University, Medford, MA 02155, USA

**Keywords:** *Caretta caretta*, corticosterone, sea turtle, stress

## Abstract

The evaluation of hormonal responses to stress in reptiles relies on acquisition of baseline corticosterone concentrations; however, the stress associated with the restraint needed to collect the blood samples can affect the results. The purpose of this study was to determine a time limit for the collection of blood samples to evaluate baseline corticosterone, haematological and biochemical results in nesting (*n* = 11) and rehabilitating (*n* = 16) loggerhead sea turtles (*Caretta caretta*)*.* Blood samples were collected from the dorsal cervical sinus of each turtle immediately after touching the animal (*t*_0_; 0–3 min) and 3 (*t*_3_; 3–6 min), 6 (*t*_6_; 6–9 min; nesting turtles only), 10 (*t*_10_; 10–13 min) and 30 min (*t*_30_; rehabilitating turtles only) after the initial hands-on time. Consistent between the rehabilitating and nesting turtles, there was a subtle yet significant increase in white blood cell counts over time. Despite the fact that white blood cell counts increased during the sampling period, there was no direct correlation between white blood cell count and corticosterone in the sampled turtles. In the nesting turtles, significant elevations in corticosterone were noted between *t*_0_ and *t*_3_ (*P* = 0.014) and between *t*_0_ and *t*_6_ (*P* = 0.022). Values at *t*_10_ were not significantly different from those at *t*_0_ (*P* = 0.102); however, there was a trend for the corticosterone values to continue to increase. These results suggest that sampling of nesting loggerhead sea turtles within 3 min of handling will provide baseline corticosterone concentrations in their natural environment. Significant elevations in corticosterone were also noted in the rehabilitating loggerhead sea turtles between *t*_0_ and *t*_10_ (*P* = 0.02) and between *t*_0_ and *t*_30_ of sampling (*P* = 0.0001). These results suggest that sampling of loggerhead sea turtles within 6 min of handling should provide baseline corticosterone concentrations in a rehabilitation setting. The delay in the corticosterone response noted in the rehabilitating turtles may be associated with the daily contact (visual or direct) they have with their human caretakers.

## Abstract (precis)

This study established a time limit for the collection of blood samples from nesting and rehabilitating loggerhead sea turtles to determine baseline corticosterone concentrations. In addition, this study assessed changes in hematologic parameters in association with the acute handling stress response in this species of sea turtle.

## Introduction

Loggerhead sea turtles (*Caretta caretta*) are a circumglobal species of marine turtle that inhabit the temperate and tropical regions of the Atlantic, Pacific and Indian Oceans. Loggerheads are the most abundant species of sea turtle found in US coastal waters, but they continue to have population declines, leading to an increased vulnerability for long-term species survival (NOAA Fisheries Office of Protected Resources, 2013). This decline is primarily attributed to fisheries-related mortalities, coastal development, increased human use of nesting beaches and pollution (NOAA Fisheries Office of Protected Resources, 2013). Due to these factors, research and conservation efforts surrounding this species are vital in order to maintain sustainable populations in the wild.

Efforts to promote recovery of the sea turtle population rely on studies of biology, physiology and ecosystem health in both free-ranging and captive populations. As coastal development, human interaction and pollution are leading to a decline in the loggerhead sea turtle population, determining how these animals cope with environmental stressors is important.

Corticosterone is the primary glucocorticoid hormone produced by reptiles in response to stressful stimuli (Gregory *et al*., 1996; Cash *et al*., 1997; Kahn *et al*., 2007; Gregory and Schmidt, 2001; Zachariah *et al*., 2009; Hunt *et al*., 2012). The evaluation of hormonal responses to stress in reptiles relies on acquiring baseline corticosterone concentrations; however, the stress associated with the capture and restraint needed to collect blood samples can be a confounding factor to the accuracy of this measure (Thaker *et al*., 2010). To minimize this effect, researchers attempt to collect blood samples within a few minutes of capture and make the assumption that this represents baseline corticosterone concentrations (Zachariah *et al*., 2009; Romero and Wikelski, 2012). Only a few studies have attempted to describe the corticosterone stress response in any chelonian species (Lance, 1994; Cash *et al*., 1997; Ott *et al*., 2000; Kahn *et al*., 2007; Zachariah *et al*., 2009), with very few studies (Gregory *et al*., 1996; Jessop *et al*., 1999, 2000, 2004a, b; Jessop and Hamann, 2004; Hunt *et al*., 2012) focused on sea turtles.

The primary goal of this study was to determine the corticosterone stress response to manual restraint in nesting and rehabilitating loggerhead sea turtles and to establish a time limit for accurate baseline corticosterone sampling in this species in these conditions. The secondary goal was to determine whether there would be any changes in haematological and biochemical parameters in association with the stress response. It was hypothesized that both nesting and rehabilitating loggerhead sea turtles would exhibit an increase in plasma corticosterone concentrations after handling procedures. It was also hypothesized that there would be an increase in the white blood cell (WBC) counts and heterophil counts during the sampling period as a result of the acute stress associated with handling.

## Materials and methods

This research was approved by the University of Illinois Institutional Animal Care and Use Committee (protocol numbers 13-072 and 13-073). An experimental field study was performed to characterize the effect of handling on corticosterone concentrations in loggerhead sea turtles over time. The study subjects represented a population of nesting and rehabilitating loggerhead sea turtles being monitored and cared for, respectively, by the Jekyll Island Authority's Georgia Sea Turtle Center (GSTC) on Jekyll Island, GA, USA. Sample size for this study was determined using the following *a priori* information: an expected increase in corticosterone of 0.3 ng/ml from baseline (*t*_0_) to any other time point, a standard deviation (SD) for this difference of 0.2, a power of 0.80 and a value of α of 0.05.

The GSTC is a state-of-the art rehabilitation, education and research centre for sea turtles and other local wildlife. The rehabilitation facilities include several hospital tanks of various sizes with sophisticated filtration systems that house injured and ill sea turtle patients. From May to early August, the GSTC staff have a nightly monitoring programme for loggerhead sea turtles that nest on the beaches of Jekyll Island.

### Sampling of nesting turtles

Sample collection from nesting loggerhead sea turtles began once the turtle had laid a minimum of five eggs or if the turtle was encountered on a false crawl. A false crawl is defined as a crawl resulting from an abandoned nesting attempt (i.e. a non-nesting crawl). Confirmation of a false crawl was made by following the tracks of the animal and confirming that no nest was present. The false crawls encountered in this study were turtles identified as already heading back to the ocean after their nesting attempt was previously aborted. These animals were restrained for sampling by standing in front of them in order to impede their progress. No restraint was required for nesting turtles, although head placement was occasionally adjusted to obtain better positioning for blood sample acquisition.

A blood sample was collected from the dorsal cervical sinus using a sodium heparinized 6 ml syringe fitted to a 20-gauge 3.8 cm needle immediately (*t*_0_; 0–3 min) after the encounter and 3 (*t*_3_; 3–6 min), 6 (*t*_6_; 6–9 min) and 10 min (*t*_10_; 10–13 min) after the initial hands-on time. The venipuncture site was disinfected using 70% ethyl alcohol. Blood samples were stored on frozen gel packs until being transported to the GSTC for processing (6–12 h).

Once the blood samples were collected, each turtle was identified using flipper tags and a passive integrated transponder tag. If the turtle did not have previous identification, a flipper tag was placed on each front flipper and a passive integrated transponder tag was placed subcutaneously in the right shoulder region. Each turtle was examined for the presence or absence of injuries. Morphometrics for each turtle were also collected, including maximal (notch to tip) curved carapace length, curved carapace width, maximal straight carapace length and straight carapace width.

### Sampling of rehabilitating turtles

Ill and injured loggerhead sea turtles were stabilized before samples were obtained for this study. The classification of a stable rehabilitating sea turtle was variable owing to the various presentations and health concerns affecting each individual. The time to stabilization varied, with most animals being stabilized within a matter of days to weeks. A turtle was classified as stable once its packed cell volume (PCV) was >15% and it was deemed likely to tolerate the sampling frequency for this study based on activity level, behaviour and eating patterns. If wounds were present on sampled rehabilitating turtles, they were stable without active infection and with normal healing in progress. The duration of rehabilitation (<2 months; >2 months) and presenting complaint were recorded for each turtle used in this study. Blood samples were obtained from the cervical sinus using the same protocol described for the nesting females, except that samples were obtained at baseline (*t*_0_; 0–3 min), 3 (*t*_3_; 3–6 min), 10 (*t*_10_; 10–13 min) and 30 min (*t*_30_; 30–33 min) after the initial hands-on time. The sampling of rehabilitating loggerhead turtles took place during routine physical examinations, during which turtles were manually restrained out of the water for the full 30 min sampling period. The degree of manual restraint varied as necessary based on the animal's activity level during the sampling period.

### Haematological and biochemical testing

Blood smears for the complete blood counts were made within 12 h of sample collection. The PCV was determined using a microhaematocrit centrifuge (TRIAC Centrifuge; Clay-Adams, Parsippany, NY, USA), and total solids were determined using a portable refractometer (Jorgenson Labs, Inc., Loveland, CO, USA). Air-dried blood-smeared slides were stained with modified Wright–Giemsa stain (HemaTek Stain Pak; Bayer Corporation, Elkhart, IN, USA) and placed in dry storage boxes. White blood cell count estimates and differentials were performed manually by the same individual (S.E.N.) using standard techniques (Fudge, 2000). Briefly, an estimated WBC count was obtained by counting the number of WBCs in 10 fields at ×400 magnification, dividing that total number by 10, and multiplying the average by 2000. The identification of WBCs for differential counts was confirmed using previously described cellular morphology for loggerhead sea turtles (Casal and Orós, 2007). Whole blood was centrifuged for 10 min at 1500***g***. The plasma was removed and frozen in a cryovial (Nalge International, Rochester, NY, USA) at −80°C until being analysed for corticosterone and plasma biochemistry.

Plasma samples for biochemical analysis were transported on dry ice to the University of Illinois (Urbana, IL, USA). Plasma biochemical analysis was processed within 6 months of collection using a portable chemistry analyser (VetScan; Abaxis Inc., Union City, CA USA). Biochemical testing was performed only on the *t*_0_ samples. Previous studies have shown that there can be some variation in the stability of plasma samples used for biochemical analysis (Williams, 2013). To minimize this risk, all samples were run at the same time to limit the likelihood of biasing the results. The following values were measured using an avian/reptile rotor (Abaxis Inc., Union City, CA USA): glucose, aspartate aminotransferase, alanine aminotransferase, alkaline phosphatase, creatinine kinase, total protein, albumin, globulin, calcium, phosphorus, sodium, potassium and uric acid.

### Corticosterone analysis

Plasma samples for corticosterone analysis were transported on dry ice to the Department of Biology, Tuft's University (Medford, MA, USA). Corticosterone samples were processed within 6 months of being collected. Previous studies have shown that corticosterone concentrations in various types of samples remain stable for multiple years while frozen at −20°C (Hunt and Wasser, 2003). The radioimmunoassay used to measure corticosterone has been described previously (Wingfield *et al*., 1992). Briefly, samples were spiked with 2000 cpm of tritiated corticosterone for later recovery analysis and allowed to equilibrate. Corticosterone was extracted from the protein component of the plasma using redistilled dichloromethane and dried using nitrogen gas. Samples were then reconstituted in phosphate-buffered saline, with an aliquot used to assess recovery. Samples were assayed in duplicate using tritiated corticosterone and a corticosterone antibody (B3-163; Esoterix, Calabasas Hills, CA, USA). Activated charcoal was used to separate the unbound from the bound steroids. The bound-to-unbound ratio was fitted to a standard curve and corrected with the recovery percentage and the original amount of plasma in order to determine the corticosterone concentration for each sample. All samples were measured in a single assay with an intra-assay variability of 2.0% and a detectability of 0.098 ng/ml.

### Statistical analysis

The distribution of the data was evaluated using the Shapiro–Wilk test, skewness, kurtosis and q-q plots. Data that were not normally distributed were log transformed for parametric testing. Normally distributed data are reported as the mean, SD and range (minimum–maximum) values, while non-normally distributed data are reported as the median, 10th–90th percentiles (%) and range values. A general linear model for repeated measures was used to determine whether corticosterone or haematological values were different over the sampling periods. Nesting status (successfully nested or not) was evaluated as a between-subject variable in the nesting loggerhead sea turtle model, while duration of rehabilitation and presenting problem were evaluated as between-subject variables in the rehabilitating loggerhead sea turtle model. Mauchly's test for sphericity was used to assess the homogeneity of covariance. If a difference was noted over time, a *post hoc* algorithm (least significant difference) was used to compare corticosterone or haematological data between specific time points. If a difference was not noted over time, the serial values were averaged for reporting purposes. Student's unpaired *t*-test was used to determine whether there was a difference in the plasma chemistry data by whether turtles nested or not. Pearson's correlation was used to determine whether corticosterone values were significantly correlated with haematological results. SPSS 22.0 (IBM Statistics, Armonk, NY, USA) was used to analyse the data. A value of *P* ≤ 0.05 was used to determine statistical significance.

## Results

### Nesting turtles

Eleven nesting loggerhead sea turtles were sampled in this study. The average maximal (notch to tip) curved carapace length, curved carapace width, maximal straight carapace length and straight carapace width were 101.9 cm (SD 13.6 cm, range 83.4–131.0 cm), 91.5 cm (SD 3.1 cm, range 84.1–94.0 cm), 90.6 cm (SD 5.8 cm, range 79.8–97.5 cm) and 71.0 cm (SD 3.7 cm, range 64.0–76.4 cm), respectively. Nine (82%) of the animals completed the nesting process during the sampling period and two (18%) false crawled.

There was a significant difference in corticosterone concentrations over time (*F* = 3.45, *P* = 0.029) in the nesting sea turtles (Table 1), with all turtles exhibiting an increase in corticosterone from baseline. Differences over time were noted between *t*_0_ and *t*_3_ (*P* = 0.014) and between *t*_0_ and *t*_6_ (*P* = 0.022). The increase in corticosterone between these two time periods was 1.3 and 1.34 times, respectively. There was no significant difference between *t*_0_ and *t*_10_ (*P* = 0.102); however, there was an increasing trend. Completing the nesting process (*F* = 0.007, *P* = 0.93) did not have a significant effect on corticosterone concentrations in this study population.

**Table 1:** Descriptive statistics for corticosterone concentrations (in nanograms per millilitre) over time in nesting loggerhead sea turtles (*Caretta caretta*; *n* = 11)

Time	Median	10–90%	Range
0 min	1.48*^,†^	0.29–3.83	0.17–4.09
3 min	1.93*	0.55–5.11	0.47–5.34
6 min	1.99^†^	0.50–4.36	0.37–4.84
10 min	2.12	0.44–5.98	0.37–6.78

^*^P = 0.014. ^†^*P* = 0.022.

There was a significant difference in WBC count over time (*F* = 3.11, *P* = 0.041) in the nesting loggerhead sea turtles, with a 1.44 times increase (*P* = 0.04) in the WBC count noted between *t*_0_ (median 1.93 × 10^3^/μl, 10–90% 1.05 × 10^3^ to 5.40 × 10^3^/μl, range 1.01 × 10^3^ to 5.40 × 10^3^/μl) and *t*_10_ (median 2.79 × 10^3^/μl, 10–90% 1.88–5.70 × 10^3^/μl, range 1.85 × 10^3^ to 5.79 × 10^3^/μl; Table 2). There was no significant difference in PCV (*F* = 0.328, *P* = 0.671), total solids (*F* = 0.612, *P* = 0.612), heterophils (*F* = 0.955, *P* = 0.427), lymphocytes (*F* = 1.19, *P* = 0.328), heterophil:lymphocyte ratio (*F* = 1.01, *P* = 0.325), monocytes (*F* = 2.25, *P* = 0.143) or eosinophils (*F* = 1.318, *P* = 0.287) over time in nesting sea turtles (Table 2).

**Table 2:** Haematological parameters in nesting loggerhead sea turtles (*C. caretta*; *n* = 11)

Parameter	Mean	Median	SD	10–90%	Range
White blood cell count (×10^3^/µl)					
*t*_0_		1.93*		1.05–5.40	1.01–5.4
*t*_3_		2.56		1.80–4.82	1.75–4.82
*t*_6_		2.52		1.32–5.17	1.2–5.3
*t*_10_		2.79*		1.88–5.70	1.85–5.79
Total solids (g/dl)	5.63		0.71		4.4–6.6
Packed cell volume (%)	35.4		5.41		28.0–44.0
Heterophils (%)	55.75		3.26		51.75–61.0
Lymphocytes (%)	29.48		6.13		23.5–39.5
Heterophil:’lymphocyte ratio	1.97		0.45		1.32–2.60
Monocytes (%)	4.59		3.76		0.75–13.5
Eosinophils (%)	9.40		4.35		4.25–18.25

*P = 0.04.

There was a significant difference in calcium status between turtles that nested and those that did not (*t* = 8.54, *P* = 0.043), with non-nesting females having significantly higher calcium concentrations (mean 15.0 mg/dl, SD 0.98 mg/dl, range 14.3–15.7 mg/dl) than nesting females (*n* = 4; mean 9.77 mg/dl, SD 1.80 mg/dl, range 7.8–11.4 mg/dl). There were no other significant differences in biochemical parameters based on nesting status (all *P* > 0.11; Table 3), and the results were consistent with previous published reports on biochemical parameters in this species (Deem *et al*., 2009).

**Table 3:** Biochemical parameters at *t*_0_ in nesting loggerhead sea turtles (*C. caretta*; *n* = 11)

Parameter	Mean	SD	Range
Aspartate aminotransferase (U/l)	155.5	44.8	86.0–213.0
Creatine kinase (U/l)	591.0^a^	297.0–2035.7^b^	297.0–5428.0
Uric acid (mg/dl)	0.95	0.44	0.5–1.70
Glucose (mg/dl)	89.2	18.6	60.0–109.0
Calcium (mg/dl)	11.5	3.1	7.8–15.7
Phosphorus (mg/dl)	8.9	1.2	7.5–10.8
Total protein (g/dl)	4.7	0.55	4.1–5.6
Albumin (g/dl)	1.4	0.21	1.2–1.7
Globulin (g/dl)	3.3	0.41	3.0–4.0
Potassium (mmol/l)	4.5	0.75	3.2–5.2
Sodium (mmol/l)	149.7	2.9	147.0–154.0

^a^Median. ^b^10–90%.

Corticosterone was not significantly correlated with any haematological or biochemical parameter (all *P* > 0.07).

### Rehabilitating turtles

Sixteen rehabilitating loggerhead sea turtles were sampled in this study. The average maximal (notch to tip) curved carapace length, curved carapace width, maximal straight carapace length and straight carapace width were 71.5 cm (SD 9.9 cm, range 56.3–90.1 cm), 67.4 cm (SD 8.9 cm, range 51.0–83.5 cm), 64.3 cm (SD 12.9 cm, range 29.6–86.0 cm) and 52.3 cm (SD 9.2 cm, range 25.5–64.5 cm), respectively. The rehabilitating turtles were presented at different ages and sizes; therefore, care should be used when interpreting these measurements. Ten (62.5%) of the animals had been at the facility for <2 months, while the remaining six (37.5%) had been there for >2 months. The sea turtles presented to the facility for debilitation (62.5%, 10/16), cold stunning (18.8%, 3/16), trauma (12.5%, 2/16) and shell deformity due to secondary nutritional hyperparathyroidism (6.3%, 1/16).

There was a significant difference in corticosterone concentrations over time (*F* = 5.8, *P* = 0.003) in the rehabilitating sea turtles (Table 4), with all turtles exhibiting an increase in corticosterone from baseline. The duration of rehabilitation (*F* = 0.01, *P* = 0.98) and presenting problem (*F* = 0.98, *P* = 0.37) did not affect corticosterone concentrations. Differences over time were noted between *t*_0_ and *t*_10_ (*P* = 0.02), *t*_0_ and *t*_30_ (*P* = 0.0001), *t*_3_ and *t*_10_ (*P* = 0.009), *t*_3_ and *t*_30_ (*P* = 0.0001) and *t*_10_ and *t*_30_ (*P* = 0.006).

**Table 4:** Descriptive statistics for corticosterone concentrations (in nanograms per millilitre) over time in rehabilitating loggerhead sea turtles (*C. caretta*; *n* = 16)

Time	Median	10–90%	Range
0 min	0.44*^,†^	0.10–7.84	0.03–12.26
3 min	0.40^‡,§^	0.09–9.08	0.07–11.47
10 min	0.82*^,‡,¶^	0.11–7.99	0.01–12.79
30 min	3.46^†,§,¶^	0.27–14.48	0.22–29.66

*P = 0.02. ^†^*P* = 0.0001. ^‡^*P* = 0.009. ^§^*P* = 0.0001. ^¶^*P* = 0.006.

There was no significant difference in PCV over time (*F* = 0.41, *P* = 0.75) or by presenting complaint (*F* = 0.063, *P* = 0.81) in the rehabilitating sea turtles; however, there was a difference in PCV according to the length of time spent in rehabilitation (*F* = 6.38, *P* = 0.025), with animals in the facility <2 months having significantly lower PCV values (mean 24.7%, SD 6.9%, range 9.7–35.7%) than those at the facility >2 months (mean 33.5%, SD 4.7%, range 26.5–38.7%; Table 5).

**Table 5:** Haematological results for rehabilitating loggerhead sea turtles (*C. caretta*; *n* = 16)

Parameter	Mean	SD	Range
White blood cell count (×10^3^/µl)			
*t*_0_	2.48	0.84	1.10–3.78
*t*_3_	2.33*	0.73	1.12–3.30
*t*_10_	2.60*	0.69	1.26–3.61
*t*_30_	2.33	0.94	1.20–3.52
Total solids (g/dl)			
Rehabilitation (<2 months)	2.81^†^	0.57	1.50–3.73
Rehabilitation (>2 months)	3.83^†^	1.16	2.03–5.28
Packed cell volume (%)			
Rehabilitation (<2 months)	24.7^‡^	6.9	9.7–35.7
Rehabilitation (>2 months)	33.5^‡^	4.7	26.5–38.7
Heterophils (%)	56.3	6.5	44.7–66.7
Lymphocytes (%)	33.1	5.8	21.7–48.0
Heterophil:lymphocyte ratio	1.78	0.49	0.96–2.92
Monocytes (%)			
Rehabilitation (<2 months)	7.5^§^	2.65	4.7–11.25
Rehabilitation (>2 months)	3.75^§^	3.29	0.0–11.25
Eosinophils (%)	3.1^a^	0.0–8.2^b^	0.0–16.5

*P = 0.006. ^†^*P* = 0.06. ^‡^*P* = 0.025. ^§^*P* = 0.04. ^a^Median. ^b^10–90%.

There was no significant difference in the concentration of total solids over time (*F* = 2.1, *P* = 0.16), by presenting complaint (*F* = 0.23, *P* = 0.63) or duration in rehabilitation (*F* = 4.03, *P* = 0.06) in the rehabilitating sea turtles; however, total solids concentrations by duration in captivity did approach significance, and sea turtles in the facility <2 months had, on average, total solids concentrations that were 26.3% lower (mean 2.81 g/dl, SD 0.57 g/dl, range 1.50–3.73 g/dl) than those that had been at the facility >2 months (mean 3.83 g/dl, SD 1.16 g/dl, range 2.03–5.28 g/dl; Table 5).

There was a significant difference in WBC count over time (*F* = 4.38, *P* = 0.03) in the rehabilitating sea turtles, with a 1.1 times rise (*P* = 0.006) in the WBC count noted between *t*_3_ (mean 2.33 × 10^3^/μl, SD 0.73 × 10^3^/μl, range 1.12 × 10^3^ to 3.30 × 10^3^/μl) and *t*_10_ sampling periods (mean 2.60 × 10^3^/μl, SD 0.69 × 10^3^/μl, range 1.26 × 10^3^ to 3.61 × 10^3^/μl; Table 5). Duration in rehabilitation (*F* = 1.91, *P* = 0.19) and presenting problem (*F* = 0.80, *P* = 0.78) did not affect the WBC count in this study population.

There was no significant difference in the monocyte counts over time (*F* = 4.12, *P* = 0.06) or by presentation (*F* = 0.56, *P* = 0.82); however, there was a difference by duration in rehabilitation (*F* = 4.77, *P* = 0.04), with animals in long-term captivity (>2 months) having higher average monocyte counts (mean 7.50%, SD 2.65%, range 4.7–11.25%) than those in rehabilitation short-term (<2 months; mean 3.75%, SD 3.29%, range 0.0–11.25%; Table 5). There was no significant difference in any of the other white blood cell types (heterophils, lymphocytes, heterophil:lymphocyte ratio and eosinophils) over time (all *P* > 0.11), by presentation (all *P* > 0.19) or duration in rehabilitation (all *P* ≥ 0.18; Table 5).

Corticosterone values were negatively correlated to WBC count (*r* = −0.661, *P* = 0.005) and lymphocyte count (*r* = −0.711, *P* = 0.02) at the 30 min sampling period. No significant correlations (all *P* > 0.10) between corticosterone and haematological values were found for any other time period. Corticosterone was positively correlated with creatine kinase (*r* = 0.898, *P* = 0.0001) and uric acid (*r* = 0.891, *P* = 0.0001) and negatively correlated with albumin (*r* = −0.926, *P* = 0.024) and sodium (*r* = −0.623, *P* = 0.031). Biochemical parameters for rehabilitating loggerhead sea turtles are listed in Table 6.

**Table 6:** Biochemical results at *t*_0_ for rehabilitating loggerhead sea turtles (*C. caretta*; *n* = 16)

Parameter	Mean	Median	SD	10–90%	Range
Aspartate aminotransferase (U/l)		294.0		116.6–781.0	97.0–959.0
Creatine kinase (U/l)		753.0		261.0–4524.8	187.0–6570.0
Uric acid (mg/dl)		0.70		0.34–2.02	0.3–2.1
Glucose (mg/dl)		114.0		85.8–166.6	83.0–175.0
Calcium (mg/dl)		6.8		5.8–9.9	5.7–10.9
Phosphorus (mg/dl)	7.4		1.0		6.1–9.2
Total protein (g/dl)	3.4		1.0		1.8–5.5
Albumin (g/dl)	1.2		0.3		0.7–1.5
Globulin (g/dl)	3.1		0.7		2.4–4.1
Potassium (mmol/l)		4.0		3.3–5.5	3.2–6.1
Sodium (mmol/l)	151.9		4.6		144.0–157.0

## Discussion

This study characterizes the response to handling stress in nesting and rehabilitating loggerhead sea turtles by the use of frequent sampling during the initial handling period. This information is vital to determining a baseline corticosterone concentration for an individual animal, and therefore, a population of animals. Studies investigating the corticosterone response in sea turtles to stressors in the wild and the potential impact that these elevated concentrations have on general health and well-being are overdue, and baseline concentrations are first required for accurate comparison. The information obtained from this study can be used as a reference for future research to evaluate the health status of an individual or population of loggerhead sea turtles and assess the effects of various stressors (e.g. environment, infectious diseases, human impacts) on their overall status.

In this study performed in loggerhead sea turtles, plasma corticosterone concentrations in response to the stress of handling were significantly increased by 10 min in a rehabilitation setting and by 3 min in a free-range setting. The most significantly elevated concentrations of corticosterone in rehabilitating loggerheads were found at the 30 min post-restraint time, and these concentrations were almost eight times that of concentrations produced at 3 min. In contrast, although nesting loggerheads showed a significant elevation in baseline corticosterone at 3 min, this increase was small in comparison to the rehabilitating loggerheads. However, it is important to note that the nesting turtles were not followed out for 30 min and that their values may have increased at a similar rate to the rehabilitating turtles. Based on these results, we suggest that baseline corticosterone concentrations can be obtained accurately from free-range nesting loggerheads within 3 min of handling and from rehabilitating loggerheads before 6 min of handling, with a preference for collecting these samples within 3 min as well.

The way in which plasma corticosterone concentrations are increased due to stress is highly variable among individuals or populations of the same species (Jessop *et al*., 1999, 2000, 2003, 2004a, b; Jessop and Hamann, 2004). Significant variation in baseline and stress concentrations of corticosterone can arise due to interactions between internal and external influences, including environmental conditions (i.e. free-ranging or rehabilitation setting), geographical location, seasonal patterns, nutritional, disease and reproductive status (Ott *et al*., 2000; Jessop *et al*., 2004a, b; Jessop and Hamann, 2004; Kahn *et al*., 2007; Zachariah *et al*., 2009; Cote *et al*., 2010; Hare and Cree, 2010; Klukowski, 2011; Trompeter and Langkilde, 2011; French *et al*., 2012; Hunt *et al*., 2012). It is possible that corticosterone responses due to handling stress may vary among the same species at different locations; therefore, the results of this study may be specific to the Jekyll Island population of loggerhead sea turtles. Analysis of baseline concentrations in different populations (including different geographical locations) of the same species is important to determine what significant differences may exist in adrenocortical function between groups.

The difference in magnitude of the corticosterone response in free-ranging vs. rehabilitating turtles is of particular interest. Adrenocortical modulation, or the ability to down-regulate the acute stress response, appears to be used in some vertebrates during periods of reproductive activity (Romero and Reed, 2005). Previous studies have suggested that the ability to produce corticosterone in response to a stressor in nesting female sea turtles is minimal or non-existent due to the need to use all available energy sources for reproductive purposes (Jessop *et al*., 1999, 2000; Jessop and Hamann, 2004). This study showed that there is a subtle yet significant rise in corticosterone in the immediate post-handling period in nesting females. In contrast, corticosterone values in the rehabilitating population rose slowly over the first 10 min of sampling and proceeded to exhibit a large spike at the 30 min sampling time. The cause of this significant increase at the 30 min sampling time is currently unknown, but would be an interesting avenue of future study in rehabilitating populations of sea turtles.

Due to the limitations of working with free-ranging wildlife, sampling was limited to four time periods per turtle in the nesting population. As the nesting period was often brief and lasted <25 min, prolonged sampling of these individuals was not feasible in most situations. It is currently unknown whether or not this population of nesting loggerheads would show a significant increase in corticosterone similar to rehabilitating turtles if sampled at 30 or 60 min post-restraint. One previous study described plasma corticosterone concentrations associated with acute captivity stress in free-ranging loggerhead sea turtles and noted a 7.2-fold increase in corticosterone after 30 min of restraint, suggesting that significant increases in corticosterone may occur at even shorter time intervals (Gregory *et al*., 1996). Unfortunately, that study did not examine corticosterone concentrations within the first few minutes of capture, and the results may have been confounded by the fact that animals were caught in nets for varying amounts of time before the initiation of manual restraint.

Investigations of the corticosterone stress response in reptiles demonstrate that there is variation in the extent of the response among species (Moore *et al*., 1991; Gregory *et al*., 1996; Cash *et al*., 1997; Gregory and Schmidt, 2001; Rostal *et al*., 2001; Jessop *et al*., 2004a, b; Al-Habsi *et al*., 2006; Deem *et al*., 2006; Ikonomopoulou *et al*., 2006; Kahn *et al*., 2007; Cote *et al*., 2010; Meylan *et al*., 2010; Klukowski, 2011; French *et al*., 2012; Hunt *et al*., 2012; Kalliokoski *et al*., 2012). Other factors that can influence corticosterone concentrations in reptiles should be taken into consideration when interpreting results. Diet, seasonal patterns, reproductive state, nutritional status, social dynamics, life stage and sex may all play a role in the adrenocortical response in these species (Zachariah *et al*., 2009; Romero and Wikelski, 2012). An attempt to minimize these variables was made by sampling all apparently healthy, reproductively active nesting females on the same beach, during the same nesting month (June). Despite these considerations, it would prove extremely challenging or impossible to control for all variables in a free-range setting.

Multiple physiological responses to a stressor are measureable, including adrenocortical, haematological and biochemical parameters. Many of these parameters have been used to determine stress levels in reptiles, including changes in haematocrit, elevations in corticosterone concentrations, changes in WBC counts and alterations in heterophil-to-lymphocyte ratios (Aguirre *et al*., 1995; Cash *et al*., 1997; Deem *et al*., 2009; French *et al*., 2012; Hunt *et al*., 2012). These parameters may provide basic information on the response of both nesting and rehabilitating sea turtles to various stressors and the correlation of those stressors with disease and immunity.

Consistent between the rehabilitating and nesting turtles, there was a subtle, yet significant increase in WBC counts over time. Changes in the leucogram (including increasing WBC counts and larger heterophil-to-lymphocyte ratios) in combination with a shift in corticosterone have been described among the physiological changes due to stress in reptiles (Aguirre *et al*., 1995). The results of the present study may suggest a similar explanation of stress-induced WBC elevations over time.

Higher plasma calcium concentrations were observed in free-ranging females that were encountered but did not initiate the nesting process. During the sampling period, the female loggerheads were encountered in various phases of the reproductive cycle. Many females sampled had already laid multiple nests in that same season, whereas others may have been encountered on their first attempt at laying. This variability in the turtle's stage of the reproductive cycle could account for the changes observed in plasma calcium concentrations in this population.

Turtles in a long-term rehabilitation setting showed a statistically significant elevation in monocytes over time when compared with turtles in short-term rehabilitation. As monocytes tend to be an indicator of chronic inflammation (Campbell, 2005), this change could correlate with these animal's long-term health problems that required an extended stay in rehabilitation for appropriate management.

Turtles in a rehabilitation setting for <2 months exhibited lower PCV and total solids concentrations when compared with turtles in rehabilitation >2 months. This finding may be consistent with the initial debilitation and anaemia often seen in sick and debilitated sea turtles (Aguirre *et al*., 1995). Previous studies in rehabilitating loggerheads have shown low PCV and total solids in animals with a variety of illnesses (malnutrition, trauma and toxin exposure), with improvement in values over time with appropriate medical therapy (Casal and Orós, 2009; Deem *et al*., 2009; Osborne *et al*., 2010).

Increased concentrations of corticosterone were correlated at *t*_0_ with increased levels of creatine kinase and uric acid in rehabilitating turtles. As creatine kinase is an indicator of muscle damage/disease and uric acid is often used as an indicator of muscle catabolism, renal function and/or dehydration in reptiles, these turtles' compromised health statuses may have contributed to an elevation in the observed corticosterone stress concentrations over time. Previous studies have shown a correlation between uric acid elevations and boat strike leading to kidney trauma as well as crude oil ingestion, both leading to renal compromise (Casal and Orós, 2009). Another study found similar elevations in uric acid in a population of rehabilitating loggerheads and attributed this increase to the debilitated state of these animals and dehydration (Deem *et al*., 2009). This same study suggested that the significantly higher levels of creatine kinase found in rehabilitating turtles were likely to be associated with muscular injury and muscle wasting in the debilitated animals (Deem *et al*., 2009). This supports a possible relationship between these chemical variables and the cause of the turtle's original presentation to the rehabilitation facility.

An interesting finding to note is the observed inverse relationship between corticosterone and albumin at *t*_0_. Albumin has been shown to be a negative acute phase protein in reptilian species. Negative acute phase proteins are those proteins whose concentration in blood decreases during an acute phase response (Paltrinieri, 2007; Eckersall and Bell, 2010). Albumin is the main negative acute phase protein, because its synthesis is decreased during an acute phase response in order to increase the amount of amino acids available for the synthesis of positive acute phase proteins during an episode of acute stress or inflammation (Paltrinieri, 2007; Eckersall and Bell, 2010). Our findings in rehabilitating turtles would be consistent with this previously reported trend.

This study provides the first characterization of an acute stress response to handling in nesting and rehabilitating loggerhead sea turtles by using more rapid serial sampling during the initial handling period. This information is useful for researchers, biologists and veterinarians in order to determine a baseline corticosterone concentration for an individual animal or population of animals. The information obtained in this study can be used for a more thorough evaluation of health status and the level of stressors affecting this protected species. Future studies on the long-term effects of elevated corticosterone in both nesting and rehabilitating sea turtles would prove beneficial.

## Funding

This work was supported by Fluker Farms (Port Allen, LA, USA) and NSF IOS-1048529 (to L.M.R.).
